# Medical Journals and Advertising

**Published:** 1892-11

**Authors:** 


### MEDICAL JOURNALS AND ADVERTISING.

The following advertisements we clip from the columns of an enterprising journal which claims to have a larger circulation than any other medical journal in the world. We submit that this is a disgrace to medical journalism which should be an exponent only of that which is conscientious, professional and honorable in thought and conduct:

FIRST UNDERSTAND, THEN SHOW YOUR APPRECIATION.—Tilghman's method to influence the sex, by which you can obtain either male or female as desired, is nature's law discovered, and is thoroughly scientific; and the author knowing this, desires to give it to the physicians of this country. The method applies to both the human and animal familes, and very many scientific stockmen are now using it with complete success and profit. Physicians, as a rule, want it for the information gained. His price is $5.00, but it will be sent to physicians for $1.00. And this is to cover expenses. Address, etc.

SECRETS OF PHYSIOLOGY.—Control of offspring without medicine. Scientific work. Price, $5.00. To physicians, $1.00. Address, etc.

It is such advertising as this that degrades the journal containing it to the level of the local newspaper. We want to say, in passing, that there is in Georgia a country newspaper, the Fort Valley *Leader*, which keeps a standing announcement on its title page that it will publish no patent medicine advertisement. But here is a *medical journal* selling its space to arrant quackery, and circulating claims which are absolutely false; just for a little money!

We do not know whether the editor of that journal is aware of it, so we will say to him that we are not yet in possession of information which will enable us to "influence the sex" or "control the offspring," however desirable such knowledge might be. But that doesn't matter.

If there is left on the title page of our esteemed St. Louis contemporary any space that is free from advertisements and large enough for a small motto, we would suggest the counsel of Iago: "*Make all the money thou canst; put money in thy purse; therefore, make money.*"

